# Does CO_2_ pneumoperitoneum in laparoscopy interfere with collagen deposition in abdominal surgical wounds?^1^

**DOI:** 10.1590/s0102-865020200060000005

**Published:** 2020-07-13

**Authors:** Pedro Henrique Alves de Morais, Rafael Francisco Alves Silva, Thiago da Silva Ribeiro, Igor Eduardo Caetano de Farias, Ruy de Souza Lino, Fabiana Pirani Carneiro, Leonardo de Castro Durães, João Batista de Sousa

**Affiliations:** I MD, General Surgeon, Universidade de Brasília (UnB), Brazil. Acquisition, analysis and interpretation of data; technical procedures; statistics analysis; manuscript writing; critical revision; final approval.; II Graduate student, Medical School, Centro Universitário de Brasília (UniCEUB), Brazil. Technical procedures, manuscript preparation, critical revision, final approval.; III Graduate student, Medical School, UniCEUB, Brasilia-DF, Brazil. Manuscript preparation, critical revision, final approval.; IV MD, General Surgeon, Irmandade da Santa Casa de Misericórdia de São Paulo, Brazil. Statistics analysis, critical revision, final approval.; V PhD, Associate Professor, Pathology Department, Institute of Tropical Pathology and Public Health, Universidade Federal de Goiás (UFG), Goiania-GO, Brazil. Histopathological examinations, critical revision, final approval.; VI PhD, Associate Professor of Pathology, School of Medicine, UnB, Brasilia-DF, Brazil. Technical procedures, histopathological examinations, critical revision, final approval.; VII PhD, Colorectal Surgery, Johns Hopkins Medicine, Baltimore, Maryland, USA. Conception and design of the study, technical procedures, critical revision, final approval.; VIII PhD, Associate Professor of Surgery, School of Medicine, UnB, Brasilia-DF, Brazil. Conception and

**Keywords:** Wound Healing, Incisional Hernia, Collagen, Pneumoperitoneum, Laparoscopy, Minimally Invasive Surgical Procedures, Rats

## Abstract

**Purpose:**

To determine by histomorphometric analysis whether CO_2_ pneumoperitoneum interferes with collagen deposition in surgical wounds in the aponeurosis of rats.

**Methods:**

This experiment involved 80 male *Wistar* rats, randomly allocated into four groups according to pneumoperitoneum period (PRE: 30 min preoperatively; POST: 30 min postoperatively; PP: 30 min pre- and postoperatively; C: control group). CO_2_ pneumoperitoneum was insufflated to 5 mmHg of pressure. A laparotomy was performed; 1 cm of the left colon was then resected, and an end-to-end anastomosis was performed to simulate surgical trauma, after which the abdominal wall was closed. On postoperative days 7 or 14, a sample of the abdominal wall was collected, stained with *picrosirius* red and observed under polarized light in an optical microscope. The amount of collagen was estimated by computerized histomorphometric analysis.

**Results:**

There were no significant differences in collagen deposition between the control and experimental groups on postoperative days 7 (p=0.720) or 14 (p=0.933). The amount of collagen increased as expected in all groups between postoperative days 7 and 14 (p=0.0003).

**Conclusion:**

At 5 mmHg, CO_2_ pneumoperitoneum does not interfere with collagen deposition in abdominal wall surgical wounds in rats.

## Introduction

Laparoscopy has been the greatest landmark in surgery since the anesthesia revolution of the 19th century^1^. Surgical laparoscopy is recent: the first gastrointestinal tract surgery by this method was a minimally invasive appendectomy performed by Kurt Semm in 1980^1,2^. The world’s first laparoscopic cholecystectomy was performed by Erich Muhe in Germany in 1985, and this surgery was first performed in Brazil by Thomaz Szego in 1990^1-5^.

Currently, laparoscopic access is the gold standard for treating a variety of comorbidities, such as cholecystitis and appendicitis^5,6^. The main advantages include faster recovery, lower surgical site infection rates, less bleeding and postoperative pain, and a quicker return to daily activities, as well as aesthetic benefits^6,7^.

To perform any laparoscopic surgery, the abdominal cavity must be insufflated with CO_2_ pneumoperitoneum to provide adequate surgical space for instrument and tissue manipulation^5-8^. Since minimally invasive surgery has only become popular in the last three decades, the safety and adequacy of its technical aspects must still be verified, including the safety of pressurized CO_2_^2-5^.

Although there are proven clinical benefits to laparoscopic procedures, insufflating the peritoneal cavity with CO_2_ alters physical and chemical factors that affect the patient’s vasculature and physiological functions^8^. The increased intraperitoneal pressure from CO_2_ pneumoperitoneum directly interferes with splanchnic microcirculation and blood flow in large vessels, which may alter renal, hepatic, cardiac and pulmonary function^9-13^. These pathophysiological changes could impair surgical wound healing^14^.

Healing is a dynamic process of three overlapping sequential stages: inflammatory, proliferative and remodeling^15^. Collagen deposition in surgical wounds occurs dynamically throughout the proliferative and remodeling stages^14-19^. Since all aspects of collagen metabolism, such as synthesis, degradation and remodeling, are related to good quality surgical scarring, it is crucial to assess this component to understand wound healing^14-19^. If CO_2_ pneumoperitoneum interferes with collagen deposition during healing, it would be a risk factor for healing complications, such as dehiscence and incisional hernia.

The objective of the present study was to determine whether CO_2_ pneumoperitoneum interferes with collagen deposition in abdominal wall surgical wounds in rats.

## Methods

This study was developed in accordance with the Universidade de Brasília Animal Care and Use Committee guidelines and approved by the ethics committee of the institution. The study also followed federal standards (no. 11.794/2008) and was reported following the Animal Research: Reporting of in Vivo Experiments (ARRIVE) guidelines.

### *Animals and surgical technique*

**Figure 1:**
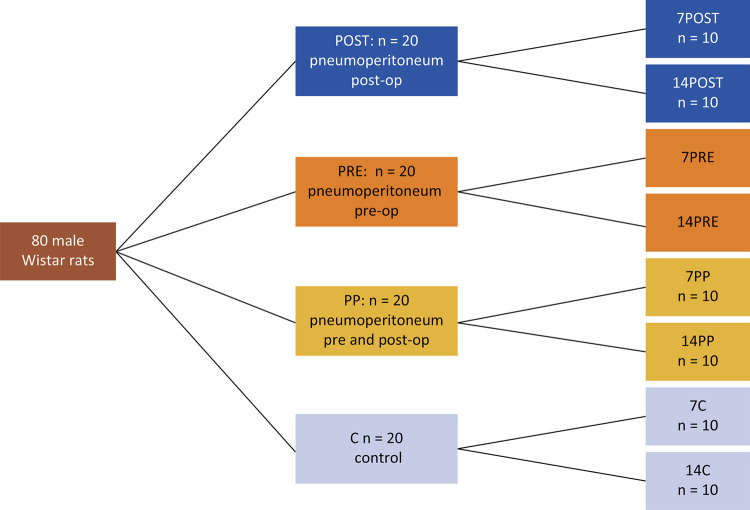
Distribution of the animals according to perioperative pneumoperitoneum time, including subgroups according to postoperative day of euthanasia.

A total of 80 male Wistar rats were randomly allocated into four groups of 20 animals (Fig. 1).

Different pneumoperitoneum strategies were adopted to simulate situations from surgery in humans:

- PRE: 30 min of pneumoperitoneum preoperatively;
- POST: 30 min of pneumoperitoneum postoperatively;
- PP: 30 min of pneumoperitoneum pre- and postoperatively;
- C: the control group underwent surgery without pneumoperitoneum.
- Animals were further divided into two subgroups according to the day of euthanasia:
- Euthanasia on postoperative day (POD) 7: 7C, 7PRE, 7POST, 7PP;
- Euthanasia on POD 14: 14C, 14PRE, 14POST, 14 PP.

It is known that prolonged exposure to pneumoperitoneum can have physiological effects, such as kidney and liver damage^8-14^. Groups exposed to 30 min (PRE and POST groups) and 60 min ( PP group) were designed to evaluate the influence of exposure on wound healing. During a laparoscopic colectomy in human patients, after dissection of the anatomical planes and ligation of the vessels by laparoscopy, an abdominal incision is made to remove the surgical specimen. The abdominal wall is then closed, and pneumoperitoneum is re-established to perform the anastomosis and hemostasis, which were simulated by the PP group. Since abdominal wall traction by pneumoperitoneum in the region of this newly made suture could impair wound healing, the POST and PRE groups were designed (both with 30 min of exposure) to isolate abdominal wall traction by pneumoperitoneum as a variable.

Anesthesia consisted of xylazine 10 mg/kg and ketamine 75 mg/kg, both intramuscularly. The abdomen was shaved, and polyvinylpyrrolidone-iodine antiseptic was applied. Pneumoperitoneum was performed with an automatic insufflator through Veress needle insertion in the lower left abdominal quadrant. CO_2_ was insufflated at a flow rate of 0.5 to 1.0 liters per minute, until 5 mmHg of pressure was established.

A 4.5-cm midline abdominal incision was made, beginning 1 cm cranial to the pubis symphysis. One cm of the left colon was then resected, and an end-to-end anastomosis was performed with single layer running sutures (6-0 polypropylene) to simulate surgical trauma. Abdominal wall closure was performed with running sutures in two layers: first the aponeurosis, then the skin (5-0 polypropylene).

On POD 7 or 14, the animals were anesthetized with an overdose of thionembutal, and a sample of the abdominal wall was collected.

### *Histomorphometric analysis*

The abdominal wall specimen was fixed in formalin and stained with picrosirius red. The amount of collagen was estimated through histomorphometric analysis.

**Figure 2:**
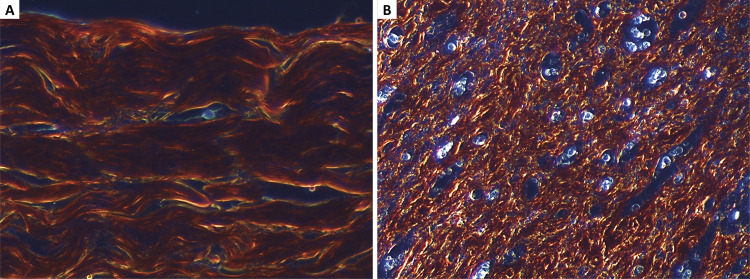
Polarized light microscopy of an abdominal cut with picrosirius coloration. Collagen type I (orange and red birefringence) in a normal area (A) and in a scar tissue area (B). (*) Blood vessel in granulation tissue.

For histomorphometric analysis, the stained sample was observed under polarized light in an optical microscope, and photographs of the surgical scar, including the entire wound, were taken. Under polarized light, collagen assumes bright colors, whereas other structures become darker (Fig. 2).

Between 25 and 30 images were obtained per case to ensure documentation of all the stained collagen in the aponeurosis, as well as the edges of the wound, including subcutaneous tissue and the abdominis rectus muscle.

These images were analyzed in ImageJ, which calculates the percentage of collagen area in each image. The final value of collagen per animal was the sum of the percentages obtained in all images.

### *Statistical analysis*

The data were analyzed in SPSS 18.0. One-way ANOVA and Student’s t-tests were performed according to the sample’s characteristics. Significance was set at p<0.05.

The sample size was based on a previous study by our group^20^ and was calculated in SPSS18.0 with a 95% confidence interval (alpha cut-off of 0.05 – probability of type I error of 5%) and study power of 80% (beta cut-off of 0.2 – probability of type II error of 20%), taking in account the treatment effect size (the expected difference between treatments was small).

## Results

There was no significant difference in collagen deposition between the control and experimental groups on POD 7 (p=0.720) (Table 1) or POD 14 (0.933) (Table 2).

**Table 1:** Number of valid cases (n) and histomorphometry values of animals killed 7 days after surgery, measured as the sum of collagen percentages obtained from all images of each animal.

	7C	7PRE	7POST	7PP	p
n	7	9	10	9	
Mean	104.9	81.6	96.3	94.8	0.72
Maximum	189.4	117.7	163.4	175.8	
Minimum	42.9	46.6	39	33.2	
Standard deviation	46.2	21.9	39.9	51.2	

**Table 2:** Number of valid cases (n) and histomorphometry values of animals killed 14 days after surgery, measured as the sum of collagen percentages obtained from all images of each animal.

	14C	14PRE	14POST	14PP	p
n	8	9	7	7	
Mean	150.4	155.9	143.5	134	0.93
Maximum	236	313.1	210	247.6	
Minimum	61.1	76.8	96	52.6	
Standard deviation	68.4	86.3	48.4	62.5	

As expected, collagen measurements significantly increased in all groups between POD 7 (mean values: 7C=104.9, 7PRE=81.6, 7POS=96.3, 7PP=94.8) (Table 1) and POD 14 (mean values: 14C=150.4, 14PRE=155.9, 14POS=143.5, 14PP=134) (p=0.0003) (Table 2).

There were 5 postoperative deaths on POD 7: 3 in group 7C, 1 in 7PRE, and 1 in 7PP. There were 8 postoperative deaths on POD 14: 2 in group 14C, 1 in 14PRE, 3 in 14POST and 3 in 14PP.

## Discussion

This study was designed to investigate the possible effects of CO_2_ pneumoperitoneum on the deposition of collagen in aponeurotic surgical wounds in rats. The animals were subjected to an intraperitoneal pressure of 5 mmHg, equivalent to that conventionally used in humans (12 to 15 mmHg)^20^.

Collagen deposition was similar in all groups on POD 7 and 14. This demonstrates that collagen deposition was not influenced by pneumoperitoneum. In a clinical setting, it means that the local repercussions of pneumoperitoneum would probably be insufficient to alter the progression of healing and would avoid complications such as aponeurosis dehiscence and incisional hernias^21-25^. Therefore, it seems reasonable to assume that that pneumoperitoneum, at the ideal pressures used in laparoscopy, is safe and validated as demonstrated in the present study as well as in previous studies performed by our group.

Collagen in scar tissue can be evaluated by several methods, and histomorphometry is the most commonly used in the literature^26^. Collagen shows strong birefringence in yellow, green and red tones when stained with picrosirius red and exposed to polarized light, making it possible to perform computerized histomorphometric analysis^20,27,28^. Healing can also be evaluated by other methods, such as hydroxyproline dosage, hematoxylin-eosin staining histopathological analysis, and tensile strength measurement^29,30^. Since collagen is critical to tissue strength and integrity and is essential in all stages of healing; its measurement in wound deposition was selected for this study^17-19,31,32^.

An environment pressurized with CO_2_ may alter angiogenesis and inflammatory response processes and, thus, interfere with the inflammatory and proliferative phases of healing^20,33^. The results of this study are consistent with those of previous studies by our group that have analyzed healing by various parameters: histopathological analysis with hematoxylin-eosin staining, tensile strength, rupture pressure, and hydroxyproline scar concentration^20,32^. In all these studies, pneumoperitoneum has proven to be safe and not to interfere with wound healing.

Morphological analysis of liver specimens under the same pneumoperitoneum conditions revealed hepatic hydropic degeneration^33^. Impaired healing can result in incisional hernias, which are the main long-term complications of open surgery and can cause pain and intestinal incarceration or strangulation^34^. Fink *et al*.^35^ found that the incidence of this type of hernia 1 and 3 years after laparotomy is 12.6% and 22.4%, respectively. Incisional hernias are caused by factors related to the patient and the surgical technique^35^. The present results corroborate those of previous studies, i.e. that CO_2_ pneumoperitoneum does not alter healing, thus reinforcing the safety of pneumoperitoneum for laparoscopy when used at adequate pressures^20,32,33^.

This study has some limitations. First, experimental data from animals are not as reliable as those from prospective studies in humans. Second, analysis of a single microscopic parameter does not necessarily represent patient outcomes in a clinical setting. Thus, prospective clinical studies are needed to clarify the results of the present study.

## Conclusion

Perioperative CO_2_ pneumoperitoneum at 5 mmHg for 30 or 60 min does not interfere with collagen deposition in abdominal surgical wounds in rats.

## Footnotes

1 Research performed at Laboratory of Experimental Surgery, Faculty of Medicine, Universidade de Brasília (UnB), Brazil.
